# An intriguing RNA species—perspectives of circularized RNA

**DOI:** 10.1007/s13238-015-0202-0

**Published:** 2015-09-08

**Authors:** Ting Shen, Miao Han, Gang Wei, Ting Ni

**Affiliations:** MOE Key Laboratory of Contemporary Anthropology & State Key Laboratory of Genetics Engineering, Collaborative Innovation Center of Genetics and Development, School of Life Sciences, Fudan University, Shanghai, 200433 China

**Keywords:** circular RNA, back splice, gene regulation

## Abstract

Circular RNAs (circRNAs), a kind of covalently closed RNA molecule, were used to be considered a type of by-products of mis-splicing events and were discovered sporadically due to the technological limits in the early years. With the great technological progress such as high-throughput next-generation sequencing, numerous circRNAs have recently been detected in many species. CircRNAs were expressed in a spatio-temporally specific manner, suggesting their regulatory functional potentials were overlooked previously. Intriguingly, some circRNAs were indeed found with critical physiological functions in certain circumstances. CircRNAs have a more stable molecular structure that can resist to exoribonuclease comparing to those linear ones, and their molecular functions include microRNA sponge, regulatory roles in transcription, mRNA traps that compete with linear splicing, templates for translation and possibly other presently unknown roles. Here, we review the discovery and characterization of circRNAs, the origination and formation mechanism, the physiological functions and the molecular roles, along with the methods for detection of circRNAs. We further look into the future and propose key questions to be answered for these magical RNA molecules.

## INTRODUCTION

CircRNA is a member of the non-coding RNA kingdom. Unlike the traditionally known RNA species, sequence of circRNA is not arranged in a normal order relative to the genomic context but arrays in a scrambled manner, in which the 3′ downstream sequences are joined to the 5′ upstream sequences, resulting in a covalent-closed circular molecule without free terminals, resemble that of plasmids.

Although circRNA was first discovered in eukaryotes two decades ago, it has just become a new research focus in recent years. Prior to the accidental experimental discovery of a few scrambled transcripts with low abundances from the *DCC* gene in the human cell line (Nigro et al., 1991), a study using an electron microscope had already reported that RNA could be present in a circular form in the eukaryotic cell cytoplasm ten years ago (Hsu and Coca-Prados, 1979). It should be noted that circRNAs can not only be the transcripts derived from a genome, but also can act as the germ plasm. Numerous viroids and viroid-like subviruses were found possessing circRNA genomes (Flores et al., 2014; Kos et al., 1986; Roossinck et al., 1992; Sanger et al., 1976; Schneider, 1969). In addition, certain genes in humans (Burd et al., 2010; Caldas et al., 1998; Cocquerelle et al., 1992; Cocquerelle et al., 1993; Li and Lytton, 1999; Surono et al., 1999; Zaphiropoulos, 1997), rats (Zaphiropoulos, 1996, 1997), mice (Capel et al., 1993), and *Drosophila* (Houseley et al., 2006), such as *ETS1* (Cocquerelle et al., 1992), *P450 2C24* (Zaphiropoulos, 1996), *Sry* (Capel et al., 1993), and *Drosophilamuscleblind* (*MBL*/*MBNL1*) (Houseley et al., 2006), were reported occasionally containing one or more circular transcripts in the past years. Due to the improvement of biochemical methods and the application of high-throughput sequencing technology, circRNAs have been widely discovered in cells and tissues of various species recently (Jeck et al., 2013; Memczak et al., 2013; Salzman et al., 2013; Salzman et al., 2012; Wang et al., 2014; Zhang et al., 2013). Therefore, circRNA is a universal RNA species and unlikely an abnormal splicing by-product or experimental artifact.

Like long non-coding RNAs (lncRNAs), the functions of circRNAs may depend on the features of their sequences and structures. Recent studies had revealed that the antisense transcript of cerebellar degeneration-related protein 1 (*CDR1as*), a circular RNA, which contained dozens of microRNA-7 (miR-7) binding sites, could resist the microRNA (miRNA)-mediated endocleavage pathway and acted as a competing endogenous molecule in gene expression regulation (Hansen et al., 2013; Memczak et al., 2013). Besides, sex-determining gene (*Sry*) circRNA could also function as a decoy to absorb miR-138 (Hansen et al., 2013). What’s more, a kind of exon-intron-derived circRNAs was able to interact with U1 snRNA based on complementary base pairing, which was advantageous for the circRNA’s parent gene expression (Li et al., 2015). Although the present understanding of the function of circRNAs is limited, their widespread existence and evolutionary conservation among species have been confirmed, suggesting that these molecules may have more regulatory functions that have yet to be revealed.

## CHARACTERISTICS OF CIRCULAR RNA

The main feature of circRNA is the intramolecular circular structure wherein the 3′ ends of some exons turns back and joins the 5′ ends of other upstream exons to form a closed molecule. Therefore, unlike those typical linear RNAs, the 5′ cap and 3′ polyadenylation tail (poly-(A) tail) are absent in these transcripts. Because of the absence of free terminals, these molecules can easily antagonize the hydrolysis by various cellular exoribonucleases. Jeck et al. proved that the half-lives of four circular RNAs exceeded 48 h, whereas the linear transcripts of corresponding parent genes (*HIPK3*, *KIAA0812*, *ASXL1*, and *LPAR1*) had half-lives of less than 20 h (Jeck et al., 2013).

Due to the low abundance, many circRNAs had just been discovered sporadically in the eukaryotic cell by using polymerase chain reaction (PCR) and usually considered to be by-products of splicing in the past a few years. For example, the abundances of circRNA derived from the *DCC* gene, took less than 0.1% of the total transcriptional level of the gene (Nigro et al., 1991). But an exceptional example was *Sry* circRNA, which was a more plentiful transcript than its linear partner in the adult testis (Capel et al., 1993). Then based on expressed sequence tag (EST) analysis, hundreds of scrambled exons were discovered, however, it failed to distinguish circRNAs from *trans*-splicing transcripts and exon repetition (Dixon, 2005; Shao et al., 2006). Benefiting from the rapid progress in next-generation sequencing technology, a huge number of these low abundant circRNA transcripts have sprung up in recent years (Jeck et al., 2013; Memczak et al., 2013; Salzman et al., 2013; Salzman et al., 2012; Wang et al., 2014; Zhang et al., 2013). It should also be noted that the abundance of some circRNAs could be over ten times as much as their linear transcript partners (Jeck et al., 2013; Salzman et al., 2013).

Nevertheless, the abundance of circRNA and the ratio between circular and linear RNA isoforms of a given gene are dynamic and often show tissue/developmental-stage-specific expression. As an example, circular RNA molecules derived from *Sry* gene are the main transcripts in embryonic brains on Days 11–19, whereas linear molecules become more ample in postnatal brains, wherein circRNAs can even be absent (Mayer et al., 2000). During the development of cell/tissue, circRNAs can authentically be modulated. When epithelial-mesenchymal transition (EMT) happened to human mammary epithelial (HMLE) cells in response to the treatment of TGF-β, hundreds of circRNAs were regulated (Conn et al., 2015). What’s more, the abundance of many circRNAs exhibited substantial fluctuation in response to synaptogenesis, consistent with the idea that circRNAs might regulate synaptic function during development (You et al., 2015). Other studies also provided evidences that circRNA expression was cell-, tissue- and developmental stage-specific (Memczak et al., 2013; Salzman et al., 2013).

CircRNAs can be derived from either exons or introns or both, and all are with great diversity in length. Most circRNAs overlap with coding exons, but some are derived from 3′ untranslated regions (3′ UTRs) and some are originated from lncRNAs (Burd et al., 2010; Memczak et al., 2013). Some introns, which are usually excised off from pre-mRNA for linear RNA, can also produce circRNAs (Memczak et al., 2013; Zhang et al., 2013). Besides, circRNA can be composed of exons and retained introns, and a parent gene can produce multiple circRNAs composed of various exons or derived from different introns (Li et al., 2015). An illustrative example is the *dystrophin* gene in humans, here, 11 circRNAs are identified in the 5′ region of this gene (Surono et al., 1999). Although most circRNAs seem to be coupled with the transcription and post-transcriptional processing of their parent genes, there exist exceptions: *Sry* circRNA is originated from the initial transcription driven by a distal promoter different from that of the linear transcript (Dolci et al., 1997), and *CDR1as* is a natural antisense long non-coding circular transcript derived from the opposite strand of *CDR1* gene (Hansen et al., 2011). The length of the circRNA can vary greatly, from hundreds to thousands of nucleotides in length. For example, a circRNA from the aforementioned *dystrophin* gene composes of two exons with the length of about two hundred bases, while another circRNA including 16 exons composed of the 2nd- to 17th-exon of the same gene exceeds 2 kilobases (kb) in length (Surono et al., 1999).

The common feature shared by all circRNAs is that they are resistant to RNase R, which digests linear RNAs (Suzuki, 2006). However, divergences exist in the structure or physiological property for circRNAs, owing to their different origin. Exon-shuffling-derived circRNA (ecRNA), including exon-intron circRNAs (ElciRNAs) consist of 3′–5′ carbon links throughout the molecule, but circular intronic RNA (ciRNA) contains a 2′–5′ link in the head-tail joint (Zhang et al., 2013), making it very sensitive or fragile to hydrolysis by debranching enzymes (Table 1). These two types of circRNAs also have other distinct sequence characteristics. For example, the canonical splice site AG/GU typically exists near the two sides of ecRNA exons, and the two introns flanking the ecRNA exons usually contain reverse-oriented repeats, which may be necessary for exon circularization (Jeck et al., 2013; Zhang et al., 2014). For ciRNA, some unique motifs exist near the 5ʹ splice site containing 7-nt GU-rich and 11-nt C-rich elements proximal to the branch point site, suggesting that these consensus motifs are critical for protecting lariat from nuclease-mediated degradation (Zhang et al., 2013). What’s more, subcellular localization for these two types of circRNAs is also different, ecRNAs are mainly present in the cytoplasm, while ciRNAs usually exist in the nucleus, indicating that they may have different functions in the cell (Jeck et al., 2013; Zhang et al., 2013).

**Table 1:** Characteristics of circRNAs

	Derivation	Localization	Joint site	Biochemical property	Sequence feature
ecRNA (Salzman et al., 2013; Memczak et al., 2013; Jeck et al., 2013; Salzman et al., 2012)	Exon	Cytoplasm	3′-5′ phosphodiester linkage	Resistant to debranching enzyme and RNase R	Long intron with reverse complementary sequences flanking the joined exons
ciRNA (Zhang et al., 2013)	Intron	Nuclear	2′-5′ phosphodiester linkage	Sensitive to debranching enzyme and resistant to RNase R	7 nt GU-rich near 5′ splice site and 11 nt C-rich in proximal to the branch point
EIciRNA (Li et al., 2015)	Exon–Intron	Nuclear	3′-5′ phosphodiester linkage	Resistant to debranching enzyme and RNase R	Long intron with reverse complementary sequences flanking the joined exons

Another property of the circRNA that should not be ignored, is the evolutionary conservation. The discoveries of circRNAs in fungi, plants, and protists indicate that circRNA is an ancient molecule tracing back over one billion years (Wang et al., 2014). In addition, circRNAs also exhibit sequence conservation. Salzman et al. showed that approximately 4% of humans and mice orthologous genes can produce circRNAs (Salzman et al., 2013). By using CircleSeq to analyze RNA-seq data generated by Salzman et al., Jeck et al. found that 44% (646/1477) of circRNAs in the murine brain were also present in the murine testis (Jeck et al., 2013). Furthermore, 457 out of 2,121 circRNAs in humans were found with circular orthologues in murine, and 69 murine circRNAs can be exactly mapped to the start and stop sites of human circRNAs (Memczak et al., 2013). The *NCX1* circular transcript was identified in humans, rats, mice, rabbits, and monkeys, indicating some circRNAs are conserved among multiple species and probably have important functions during evolution (Li and Lytton, 1999). Based on genome-wide analysis, coding-region-derived circRNAs exhibit high universal sequence conservation and more significant conservation in the third position of genetic codon (Memczak et al., 2013). Additionally, for intergenic and intronic circRNAs, the conservation is moderate, but they still exhibit a remarkable enrichment of conserved nucleotides (Memczak et al., 2013).

### Mechanism of circular RNA formation

Because circRNAs are usually coupled with the transcription of their parent genes, their dissociation from linear transcripts may be associated with nascent RNA processing, and splicing may be the event driving dissociation. Previous studies have shown that circRNA formation is interrelated with splicing, for example, *ETS1* circRNA is considered a mis-splicing product that occurs during exon skipping (Cocquerelle et al., 1993). Furthermore, the junction sites of the circRNA identified among various eukaryotic species are mostly flanked by canonical splice sites, the 5′ donor site GU and 3′ acceptor site AG, caused by a splicing event called the “back splice” (Jeck et al., 2013).

As mentioned above, biogenesis of circRNAs usually accompanies with the transcription and splicing of the parent gene, suggesting that their formation may compete with linear splicing. Splicing efficiency had been reported to have an effect on whether an exon is spliced into a linear or circular transcript (Ashwal-Fluss et al., 2014). Efficient splicing may promote mature linear RNA formation, otherwise antagonize this process. RNA polymerase II (Pol II) might be involved in this event, since several studies have shown evidence that the Pol II elongation rate is antagonistic to splicing efficiency (de la Mata et al., 2003; Ip et al., 2011; Khodor et al., 2011). It can be supposed that Pol II moving along DNA and extending RNA strand at an increasing speed would decrease the splicing efficiency. Therefore, the fast-moving Pol II could potentially provide an opportunity for some responsible *trans*-acting factors to recognize the exposed binding sites within the retained introns, and lead the dispersed introns to approach each other to generate circRNAs.

Some linear RNAs and circRNAs can be mutually exclusively transcribed. In this scenario, independently transcribed pre-mRNA serves as the precursor of circRNA, similar to that of miRNA. For example, the promoter of the *Sry* circRNA transcript differs from that of canonical linear transcript. The alternative promoter of the *Sry* gene produces a precursor transcript with long reverse-orientated repeats at the 5′ and 3′ arms, which may facilitate the *Sry* circRNA formation (Dolci et al., 1997). Another example is *CDR1as* circRNA, which is derived from the minus strand of *CDR1* gene and transcribed at opposite direction (Hansen et al., 2011). It then undergoes backsplice event to produce circular molecules during RNA processing.

Since not all exons of a gene can form circRNAs, some elements within the gene may promote to determine which exons are circularized. Introns, particularly the large ones adjacent to the joined exons tend to be involved in the backsplice. Some sequence features are favorable for circRNAs formation, though it is not clear how exons far apart come close to each other. Repeat elements (such as *ALU*) in inverted orientation within the introns flanking the joined exons are indispensable *cis-* elements, and they are reported contributing to the circularization of RNA (Jeck et al., 2013; Zhang et al., 2014). In human, the amount of *ALU* repeats within introns adjacent to the circularized exons is twofold as that in the introns flanking linear exons, and the pairs of *ALU* sequences in the flanking introns tend to be complementary (Jeck et al., 2013). Repetitive sequences are dispersed and can amount to nearly half of the genome, and *ALU* sequences are the most abundant repeat elements and reside mainly within introns (Batzer and Deininger, 2002; Chen and Sarnow, 1995; Price et al., 2004; Waterston et al., 2002). This feature increases the possibility that larger introns contain more repeat elements than smaller introns, and thus, larger introns could facilitate the circularization of adjacent exons. In addition, circularized exons tend to be skipped exons in the linear isoforms (Surono et al., 1999; Zaphiropoulos, 1996, 1997). Moreover, one of the features of skipped exons is flanked by longer introns, and there is evidence supporting that complementary motif sequences in the adjacent introns are associated with exon skipping (Keren et al., 2010).

Many proteins are most likely to be involved in this process and may also help determine which exons would undergo backsplice. A study has confirmed that spliceosome participated in this process (Ashwal-Fluss et al., 2014), in which proteins recognize sequence elements in flanking introns or exons, recruit other proteins, such as U1 snRNP, hnRNP proteins, and SR proteins, thus change RNA conformation and cause to-be-joined exons to approach each other (Kramer, 1996). *Mouseblind* (*MBL*/*MBNL1*), a splicing factor, is thought to promote its own circRNA biogenesis from the second exon in *Drosophila* and humans (Ashwal-Fluss et al., 2014). Both the flanking introns and second exon contain predicted MBL-binding sites, and the MBL levels could have a considerable effect on circRNA formation. However, the sites within the flanking introns affect MBL binding more than those in the exons do. Furthermore, MBL may bind to an intron to drive both sides of the intron approaching and to form a favorable structure for the backsplice (Ashwal-Fluss et al., 2014). Because circRNA production is tissue and developmental stage specific, the dynamic expression of some *trans-*acting proteins may be associated with such specificity. Intriguingly, Quaking (QKI) protein, an RNA binding protein containing KH domain and also an alternative splicing factor, was found to be correlate well with the circRNAs dynamic change during EMT, wherein, QKI protein recognizes the QKI binding motifs within the introns adjacent to the joined exons of circRNAs to promote the circRNAs biogenesis (Conn et al., 2015).

Various models trying to explain the circRNA generation mechanism have been proposed. The exon-skipping-circRNA hypothesis was proposed first based on the observation that the circRNA derived from *PC450 2C24* gene are correlated with related exon skipping. The author put forward two models to explain the hypothesis. The first model is inverse splicing, in which the downstream exon turns around to become proximal to the upstream exon and ready for splicing, resulting in a circular and exon-skipped linear transcript. The second model is lariat splicing, which produces alternatively spliced RNA and a lariat intermediate, and then the introns in the lariat are removed as the canonical-splicing process (Zaphiropoulos, 1996). However, recent evidence has indicated that exon skipping is not necessarily associated with circRNA formation. For example, alternatively spliced transcripts of the *dystrophin* gene can be detected, but the corresponding circRNA is absent (Surono et al., 1999). Recently, several studies have proposed two other revised models based on additional data. The first is the lariat-driven model, which is consistent with lariat splicing and predicts the co-occurrence of circRNA and exon skipping. The second is the intron-pairing-driven model, which emphasizes that flanking inverted repeats aside back-spliced exons are crucial for the direct splicing and ligation between the downstream donor site and the upstream acceptor site (Jeck et al., 2013; Jeck and Sharpless, 2014; Zhang et al., 2014). However, which model is preferentially adopted in a certain gene or cell is still unknown at present.

## BIOLOGICAL FUNCTIONS OF CIRCULAR RNAs

**Figure 1:**
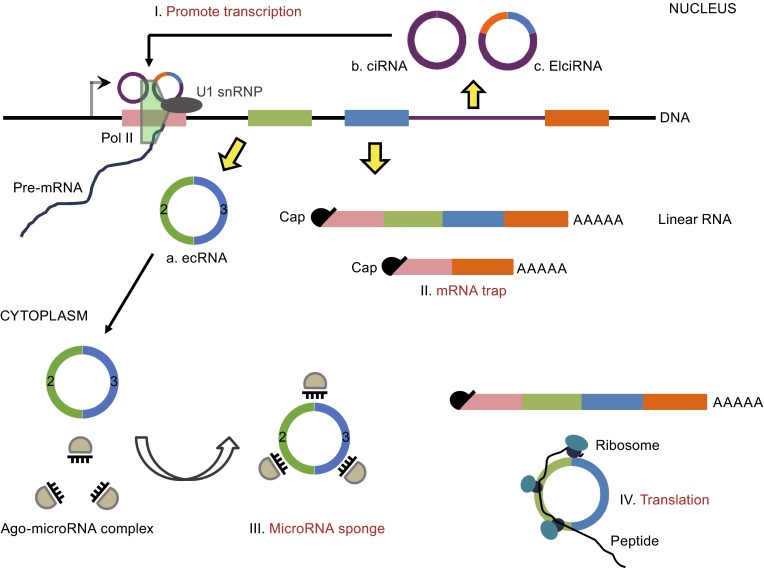
Circular RNA formation and function. CircRNAs can be categorized into three classes based on their origination. Here, exons of the gene model is illustrated with rectangle highlighted with different colors and introns is presented by thin lines, and transcriptional start site is drawn as right-angled arrow. Exon-shuffling-derived circRNA (ecRNA) is only comprised of exons (A), and circularization of intron forms another kind of circular RNA—circular intronic RNA (ciRNA) (B). The third categorization is elciRNAs which is made up of exon and retained intron (C). Four potential biological functions of circRNAs have been suggested. I. Promote transcription. CiRNAs and elciRNAs are retained in the nuclear and they can interact with transcription machinery (Pol II shown by green pentagon, U1 snRNP depicted as oval) to promote their parent gene expression. II. mRNA trap. The biogenesis of circRNAs is usually coupled with transcription and processing of their parent gene. Therefore, circRNA may negatively regulate the abundance of mature linear RNA (described by four colored rectangle with 5′ cap and 3’ polyadenylation tail (poly-(A) tail) to some extent. III. MicroRNA sponge. EcRNAs locating in the cytoplasm can antagonize microRNA-mediated endocleavage pathway (Ago-microRNA complexes presented by semicircle and comb) and function as competing endogenous molecules of microRNA. IV. Translation. EcRNAs containing the internal ribosome entry site (IRES) can be bound by ribosomes (shown by two closely-combined oval) and translated from the AUG start codon

Recent investigations have suggested four possible biological functions of circRNAs (Fig. 1); specifically, they can serve as microRNA sponges (Hansen et al., 2013; Memczak et al., 2013), promoting transcription of parent gene (Li et al., 2015; Zhang et al., 2013), mRNA traps (competing with linear splicing) (Ashwal-Fluss et al., 2014; Chao et al., 1998), and templates for translation (Surono et al., 1999; Wang and Wang, 2014).

Because of the stability of the circular structure and the microRNA-binding capacity for some circRNAs, circRNA is an ideal molecule to serve as a microRNA sponge (Fig. 1, function III), as confirmed in *CDR1as* and *Sry* (Hansen et al., 2013). *CDR1as* circRNA is a competing endogenous RNA containing more than 70 miRNA target sites for miR-7. The abundance of miR-7 targeted transcripts increased when *CDR1as* circRNA was over-expressed in HEK293 cell line, similar to the effects observed after miR-7 knockdown (Hansen et al., 2013; Memczak et al., 2013). Furthermore, a mammalian *CDR1as* circRNA over-expression vector was injected into zebrafish embryos with the genetic background of normal expression of conserved miR-7 but depletion of the *CDR1* gene locus in the embryonic brain (Kapsimali et al., 2007). The results demonstrated an obvious reduction in midbrain size of the embryos, consistent with the observation when miR-7 morpholino was injected into the zebrafish embryos (Memczak et al., 2013). In addition, *Sry* circRNA is also assumed to be an antagonist of miR-138 (Hansen et al., 2013). Since that some circRNAs contained one or more types of miRNA seed sequences based on the sequences analysis, the association of miRNAs with diseases indicated that circRNAs may have a regulatory role in the development of certain diseases (Ghosal et al., 2013). For example, miR-7 is related to numerous diseases, such as Parkinson diseases (Junn et al., 2009), diabetes (Wang et al., 2013), and cancer (Kefas et al., 2008; Saydam et al., 2011; Webster et al., 2009), raising the possibility that *CDR1as* circRNA might also be involved in these diseases by sequestering miR-7. However, most circRNAs have less than 10 miRNA binding sites (Jeck and Sharpless, 2014) and may not be effective miRNA sponges. Moreover, circRNA holds potential to function through other means, such as recruiting protein as a storage pool or transport, similar to the role of lncRNA in regulating networks or removing excessive proteins.

Considering the correlation between circRNAs and their parent gene transcription, circRNA may affect parent gene expression through *cis*- or *trans*- actions. Intron-derived circRNAs (ciRNA) retained in the nucleus can act as activators of parent gene by interacting with Pol II machinery (Fig. 1, function I) (Li et al., 2015; Zhang et al., 2013). Besides, EIciRNAs (exon-intron circRNAs) have analogous effects to parent gene transcription by interacting with U1 snRNP and Pol II through complementary sequence pairing between EIciRNA and U1 snRNA (Fig. 1, function I) (Li et al., 2015). What’s more, the modulation of the relative abundance between some ecRNA and the protein of its linear partner can resemble (in some degree) the universal positive feedback in gene regulation network. For example, though the MBL protein is required for its congenetic circRNA production by binding to corresponding element in the exon and intron of the pre-mRNA, the resulted circRNA can also recruit MBL to remove redundant cellular proteins (Ashwal-Fluss et al., 2014).

However, negative correlation is also found between formation of some ecRNA and their mature linear RNA, possibly caused by competition between forward splicing and reverse splicing (or back splice). It is assumed that backsplice produces circular RNA and corresponding truncated linear transcript (Fig. 1, function II), which is unable to translate, reducing the functional protein level as a result. The phenomenon that circRNA formation competed with productive linear mRNA was called mRNA trap (Fig. 1, function II) (Chao et al., 1998). Although in the mRNA trap model, the major role of circRNA is the linear splicing competition, one cannot rule out certain function by circular RNA itself. Take *fmn* gene as an example, mutations of the acceptor site in the fourth or fifth exon of the *fmn* gene lead to the depletion of corresponding ecRNA, but did not affect on the productive (or translatable) linear RNA. Further study demonstrated that this specific *fmn* mutant mice exhibited an incomplete penetrant renal agenesis phenotype, suggesting that the unproductive circRNA could have important physiological roles in regulating developmental process (Chao et al., 1998; Kramer, 1996), though the molecular mechanism remains elusive.

Although many circRNAs overlap with coding exons, the translation ability of circRNA remains debated (Fig. 1, function IV). Some studies have shown that circRNAs containing the internal ribosome entry site (IRES) can be translated from AUG start codon (Chen and Sarnow, 1995; Wang and Wang, 2014), while another study indicated that circRNAs cannot be accessed by ribosomes (Jeck et al., 2013). It is speculated that the absence of a 5′ cap and 3′ poly-(A) tail limits translation initiation. However, opposite arguments exist that the circular structure can be beneficial for ribosome recycling once translation initiated, which would facilitate producing more proteins than linear RNA (Perriman and Ares, 1998). Intriguingly, a truncated protein was obtained from a circRNA of the Na/Ca exchanger gene 1 (*NCX1*) in transfected cells. The size of the truncated protein was consistent with the predicted molecular weight (MW) of approximately 70 kDa, and also exhibited Na/Ca exchange activity (Li and Lytton, 1999). Although the author could not identify the same-sized proteins in native tissue, a distinguished and slightly smaller band could be observed, suggesting that this protein may be the hydrolysis residual of the circRNA-derived protein (Li and Lytton, 1999). Additionally, Wang et al. evidenced that plasmid-derived circRNA can produce a GFP protein, if IRES was introduced before the open reading frame of the *GFP* gene (Wang and Wang, 2014). Another example that circRNA can be translated into a protein in a mammalian cell was the single stranded circular RNA genome of the hepatitis δ virus, which was a satellite virus of the hepatitis B virus (Kos et al., 1986). Thus, some circRNAs could have translation potential, which might be triggered under certain conditions.

## METHODS FOR IDENTIFYING AND VALIDATING CIRCULAR RNAs

Due to its circular structure, the migration rate of circRNA is different from that of linear, interlocked, and lariat RNAs in eletrophoresis (Hansen et al., 2013; Tabak et al., 1988). CircRNA can also be characterized through Gel trapping since it can attach to a well when mixed with melted agarose due to its circular structure (Schindler et al., 1982). The more credible way to recognize circRNA for a specific gene is using PCR with out-facing primer pair (located near the back-splicing sites on the corresponding linear transcript), though false-positive cannot be excluded because of various reasons. Both genome rearrangement and *trans*-splicing can produce exon shuffling transcripts, and dislocated exons produced by template switch in *in vitro* reverse transcription (RT) is another artifact in circRNA detection using RT-PCR. Therefore, more accurate methods are required to confirm the positive results, such as using the same out-facing primer pair for the genomic DNA as a control when PCR was carried out. There are optional ways to improve the accuracy in identifying circRNA, such as enriching circRNA by degrading linear RNA with exonuclease—RNase R or tobacco acid pyrophosphatase combined with 5′-phosphate-dependent exonuclease (Hansen et al., 2011b). Northern blotting and RNase protection assay can also serve as complementary methods for validating circRNAs.

The extremely low abundance of circRNA is a constraint for its PCR-based detection. High-throughput sequencing combined with bioinformatic analysis has greatly facilitated the discovery of this kind of RNA molecule (Table 2). Unlike the normal RNA sequencing library preparation step, disposing samples through methods such as ribosomal RNA depletion and poly (A) minus RNA selection alone or in combination with RNase R treatment is indispensable prior to library construction, which has favored the discovery of circRNAs (Danan et al., 2012; Jeck et al., 2013; Zhang et al., 2013).

**Table 2:** Discovery of circRNAs by high-throughput RNA sequencing

Cell types	Methods of RNA-seq library construction	Scrambled transcripts	Strategies for identification of circRNAs	References
Human CD19^+^ B cells, CD34^+^ stem cells and neutrophils	Ribosomal RNA-depleted paired-end RNA-seq	Comprising ~10% transcripts from more than 800 genes	Based on annotated exons and utility of paired-end RNA-seq data property	Salzman et al. (2012)
15 cell types, poly-(A) minus RNA-seq data from ENCODE project	Ribosomal RNA-depleted paired-end RNA-seq	46,866 intragenic splice junctions in 8466 genes	Based on annotated exons and utility of pair-end RNA-seq data property	Salzman et al. (2013)
Human cell line Hs68 and Jurkat E6-1	Ribosomal RNA-depleted paired-end RNA-seq combined with digestion of RNase R	25,166 backsplice events, representing ~14.4% activated transcribed genes in human fibrolasts	CircleSeq	Jeck et al. (2013)
Human CD19^+^, CD34^+^, neutrophils and HEK293; Mouse brains, fetal head and differentiation-induced embryonic stem cells; *C. elegans*: oocyte, 1-cell embryo and 2-cell embryo	Ribosomal RNA-depleted paired-end RNA-seq	1950 circRNAs in human, 1903 circRNAs in mouse and 724 circRNAs in nematode	Based on splice sites and annotated transcripts	Memczak et al. (2013)
Human stem cell line H9	Poly-(A) minus and ribo-depleted and RNase R digested RNA-seq	103 circular intronic RNAs	Based on alignment to annotated human RefSeq databases	Zhang et al. (2013)
Fungi, *Arabidopsis thaliana*, and protists	Ribosomal RNA-depleted paired-end RNA-seq		Based on annotated exons and utility of paired-end RNA-seq data property	Wang et al. (2014)
Human stem cell line H9	Poly-(A) minus and ribo-depleted and RNase R digested RNA-seq	9639 exonic circular RNA	Based on alignment to annotated human RefSeq databases	Zhang et al.(2014)
Human cell lines HeLa, HEK293	Pol II CLIP followed by RNA sequencing	111 circRNAs with intron ‘retained’, termed exon–intron cicrRNAs or EIciRNAs	Pol II CLIP followed by 80-nt single-end RNA sequencing	Li et al. (2015)

To exclude confounding interferences and obtain convincing results, various algorithms have been developed to analyze high-throughput sequencing data. On the basis of the sequence order or any abnormal exon–exon junction boundaries from annotated exon, scrambled RNA molecules have been distinguished from linear molecules (Memczak et al., 2013; Salzman et al., 2013). Reads aligned to the known spliced junctions are considered as linear splicing and thus are filtered out, while reads that only span the 3′ end of the downstream exon and 5′ end of the upstream exon are considered potential signals of circRNA, in which the junction is AG/GU but not the canonical GU/AG (Memczak et al., 2013). In addition, other innovative strategy or bioinformatic analysis methods can also be applied to identify novel circRNAs (Salzman et al., 2013).

Lastly, to detect low-abundance circRNAs, RNase R nucleases were used to enrich circRNA by eliminating linear RNA prior to RNA-seq library construction (Jeck et al., 2013; Zhang et al., 2013). Mapping algorithms dependent (e.g., MapSplice (Jeck et al., 2013; Wang et al., 2010)) or independent on gene annotation (e.g., TopHat-Fusion (Zhang et al., 2013)) could be used to detect the potential junctions of circRNAs. Experimental validation was followed to confirm the existence of identified circRNAs.

## CONCLUSION AND PERSPECTIVE

Recently, the discovery of circRNAs derived from thousands of genes had disclosed a new layer of post-transcriptional regulation in many species, and greatly expanded our knowledge in understanding the complexity of gene regulation. Increasing studies are continuing to prove the universal presence and evolutionary conservation of circRNA. However, there are still some important aspects that future studies should focus on.

The function and physiological/pathological relevance of most circRNAs remained to be elucidated, although a few studies had shown promising roles of circRNAs, such as miRNA sponge, promoting transcription of parent gene, mRNA traps, and translation templates. Linear lncRNAs can act as a scaffold to recruit proteins depending on their sequence or structure. It is thus possible that circRNAs, as a special type of lncRNA, can also hold the potential to function in a similar way. CircRNAs had been widely identified in many species or special context such as developing stages or clinical samples, but their physiological/pathological roles remained elusive. Further intensive studies are warranted to explore the molecular and biological functions of circRNAs.

The biogenesis mechanism of circRNAs is under intensive investigation recently. Multiple lines of evidence supported the hypothesis that complementary-sequence-mediated exon circularization could be a broad source of circRNA production (Jeck et al., 2013; Zhang et al., 2014). However, most studies illustrating the mechanism are based on exogenous plasmid transient infection rather than manipulate corresponding elements in the host genome by genome editing strategy such as CRISPR/Cas9. In addition, *trans*-acting factors involved in circRNA biogenesis and those determine their tissue/stage/environment specific expression are urgently to be identified for fully understanding the molecular mechanism by which circRNAs are generated and regulated. Promisingly, some RNA binding proteins (RBPs) with dynamic expression changes were supposed to be involved in the circRNA biogenesis, such as QKI protein (Conn et al., 2015). It is expected that the list of responsible RBPs will show a growing increase in the next few years. Similar to the idea that RBPs can regulate alternative splicing and alternative polyadenylation, the local concentration of RBPs also holds the potential to affect the alternative circularization in circRNA formation, and may eventually contribute to the tissue/stage/environment specific expression of circular RNAs.

Moreover, future studies should take into consideration of circRNA degradation or turn-over, since both degradation and biogenesis of circRNA determine their temporal-spatial specific accumulation, and contribute to their biological function in certain circumstances. However, it is almost totally unknown in this important aspect for circRNAs, though a conformed evidence has shown that a circRNA, *CDR1as*, can be negatively regulated by miR-671 through Ago2-mediated endocleavage pathway (Hansen et al., 2011). Identification of factors such as ribonucleases responsible for the degradation of circular RNAs in the cell may be a key and fundamental question.

With the continuous decrease of sequencing cost, RNA-seq become the most prevalent method for identification of circRNAs. Although a few bioinformatic algorithms such as MapSplice (Wang et al., 2010), TopHat-Fusion (Kim and Salzberg, 2011), and CIRI (Gao et al., 2015) were developed for global discovery of circular RNAs, the consistency of these computational tools remains relatively low. RNase R treatment largely removes the false positive results, however, this strategy also impairs the quantitative feature of the circRNAs, especially the co-expression correlation between circRNAs and their parent genes. Furthermore, how to accurately quantify the expression of circRNAs is still a challenge since existing methods only count the junction reads, which are easily affected by multiple factors such as sequencing depth, read length, and fragmentation condition during RNA-seq library construction. A comprehensive comparison of existing computational methods and developing more robust and accurate bioinformatic tools will definitely move this field forward.

Lastly, future studies should also attach some importance to circRNA-involved regulatory network, which would help us comprehensively understand the formation, biological and physiological function, and degradation of circRNAs. To sum up, circRNA, acting as a new transcriptome regulation player, is attracting an increasing number of researchers to this emerging field.

## ABBREVIATIONS

circRNAs: circular RNAs
ciRNA: circular intronic RNA
ecRNA: exon-shuffling-derived circRNA
ElciRNAs: exon–intron circRNAs
EMT: epithelial–mesenchymal transition
EST: expressed sequence tag
lncRNAs: long non-coding RNAs
miRNA: microRNA
PCR: polymerase chain reaction
